# Drebrin controls neuronal migration through the formation and alignment of the leading process

**DOI:** 10.1016/j.mcn.2012.01.006

**Published:** 2012-03

**Authors:** Xin-peng Dun, Tiago Bandeira de Lima, James Allen, Sara Geraldo, Phillip Gordon-Weeks, John K. Chilton

**Affiliations:** a Institute of Biomedical and Clinical Science, Peninsula Medical School, University of Exeter, Research Way, Plymouth PL6 8BU, UK; b MRC Centre for Developmental Neurobiology, King's College London, New Hunts House, Guys Campus, London Bridge SE1 1UL, UK

**Keywords:** OMN, oculomotor nucleus, PCN, precerebellar nuclei, YFP, yellow fluorescent protein, Drebrin, Actin-binding, Migration, Leading process, Oculomotor

## Abstract

Formation of a functional nervous system requires neurons to migrate to the correct place within the developing brain. Tangentially migrating neurons are guided by a leading process which extends towards the target and is followed by the cell body. How environmental cues are coupled to specific cytoskeletal changes to produce and guide leading process growth is unknown. One such cytoskeletal modulator is drebrin, an actin-binding protein known to induce protrusions in many cell types and be important for regulating neuronal morphology.

Using the migration of oculomotor neurons as a model, we have shown that drebrin is necessary for the generation and guidance of the leading process. In the absence of drebrin, leading processes are not formed and cells fail to migrate although axon growth and pathfinding appear grossly unaffected. Conversely, when levels of drebrin are elevated the leading processes turn away from their target and as a result the motor neuron cell bodies move along abnormal paths within the brain. The aberrant trajectories were highly reproducible suggesting that drebrin is required to interpret specific guidance cues. The axons and growth cones of these neurons display morphological changes, particularly increased branching and filopodial number but despite this they extend along normal developmental pathways.

Collectively these results show that drebrin is initially necessary for the formation of a leading process and subsequently for this to respond to navigational signals and grow in the correct direction. Furthermore, we have shown that the actions of drebrin can be segregated within individual motor neurons to direct their migration independently of axon guidance.

## Introduction

Neuronal migration is an essential feature of brain development and occurs in two main modes. Radially migrating neurons move along an existing scaffold of glial cells away from the neuroepithelium. In contrast, tangentially migrating neurons navigate their way through the developing brain by extending a leading process towards their target which the cell body subsequently follows (Marin et al., 2010; Metin et al., 2008). The leading process is morphologically similar to an axon and several axon guidance factors are known to direct the trajectory of migration (Cubedo et al., 2009; Marcos et al., 2009; Renaud et al., 2008; Zhu et al., 2009). Indeed, in many neurons, such as those of the precerebellar nuclei (PCN), the leading process becomes the axon once migration has finished (Bloch-Gallego et al., 2005). In other instances, such as the midbrain oculomotor nucleus, it is distinct from the axon and is believed to be a transient feature required for migration (Chilton and Guthrie, 2004; Puelles-Lobez et al., 1975; Puelles, 1978). In both tangentially and radially migrating cells, the actin and microtubule networks combine to provide the motile force (Bellion et al., 2005; Schaar and McConnell, 2005; Tsai et al., 2007). The means by which these cytoskeletal elements are regulated to direct leading process growth and whether this differs between modes of migration is unknown.

**Fig. 1:**
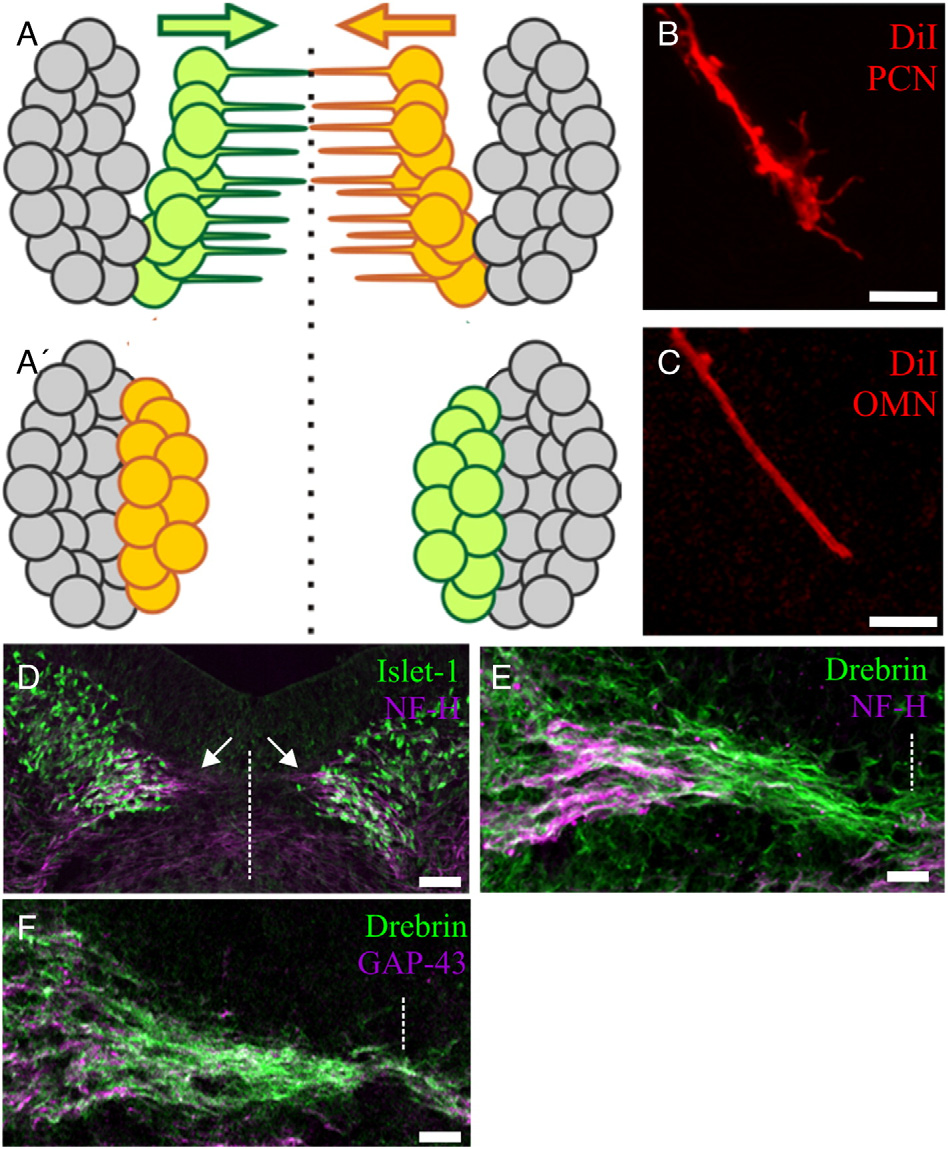
Molecular characterisation of oculomotor leading processes. (A) Schematic longitudinal view of oculomotor migration. The ventromedial subnuclei on each side (green/orange) extend leading processes to the midline (A) the cell bodies follow and swap places (A′). (B) DiI labelling of precerebellar leading process and (C) oculomotor leading process. (D) Transverse cryosection of HH stage 29 chick midbrain showing oculomotor cell bodies immunolabelled with Islet-1 (green) and their proximal leading processes (arrows) with neurofilament heavy chain (NF-H, purple). Higher magnification of leading processes labelled with (E) NF-H (purple) and drebrin (green) and (F) GAP-43 (purple) and drebrin (green). Dashed line indicates midline, scale bar = 25 μm (B,C,E and F), 100 μm (D).

The developing oculomotor nucleus (OMN) is a good model system to study tangential migration because it involves a discrete population of motor neurons in a well-defined and accessible location. The OMN is divided into four subnuclei, each innervating one of the extraocular muscles that rotate the eyeball. The ventromedial subnucleus displays unusual behaviour for motor neurons in that these cells extend a leading process across the midline which the cell body subsequently follows (Fig. 1A); the axon is a distinct process and remains behind growing towards the target muscle. The result is that the ventromedial subnuclei swap positions and end up projecting contralaterally (Chilton and Guthrie, 2004; Heaton and Wayne, 1983; Puelles-Lobez et al., 1975).

Microtubule-actin interactions have been intensively studied in axonal growth cone dynamics (Geraldo and Gordon-Weeks, 2009) and the emerging knowledge of their molecular regulation may be extrapolated to shed light on the control of neuronal migration. One such factor is the actin-binding protein drebrin which is involved in controlling the morphology and motility of cells in a range of tissues (Dun and Chilton, 2010; Shirao et al., 1990, 1992). Overexpression of drebrin in hippocampal neurons leads to the formation of abnormally large spines (Mizui et al., 2005), conversely loss of drebrin results in sparse, thin spines (Takahashi et al., 2006) and reduced drebrin levels in humans are associated with Alzheimer Disease (Calon et al., 2004; Shim and Lubec, 2002). Drebrin is enriched within the transition zone and filopodia of growth cones where it binds to F-actin bundles. Within the filopodia it also binds EB3, a microtubule plus-end protein; this interaction is required for the formation of growth cones and neurites (Geraldo et al., 2008).

Here we show that functionally manipulating drebrin has specific effects on neuronal migration by perturbing the generation and orientation of leading processes. We have characterised the leading process of migrating oculomotor neurons and demonstrated that drebrin is required for the formation of a leading process and subsequently regulates its pathfinding behaviour. Using the unique properties of this population of motor neurons we have been able to distinguish a separate effect of drebrin in axons. Up or downregulation of drebrin function strongly affects the morphology of oculomotor axonal growth cones yet their pathfinding abilities are relatively unimpaired. Our data reveal the functional compartmentalisation of an actin-binding protein within individual neurons to regulate their migratory pathway independently of axon guidance.

## Results

### Molecular and morphological characterisation of oculomotor neuron leading processes

In many tangentially migrating neurons, such as those of the PCN, the leading process eventually becomes the axon (Bloch-Gallego et al., 2005). Oculomotor neurons are different; the axon is a distinct process which extends out into the periphery, the leading process projects within the midbrain to the contralateral nucleus (Chilton and Guthrie, 2004; Puelles-Lobez et al., 1975). We used 1,1′-dioctadecyl-3,3,3′3′-tetramethylindocarbocyanine perchlorate (DiI) labelling to compare the morphology of oculomotor and PCN leading processes. At HH stage 26, well before they reach the cerebellum, the PCN processes resemble axons with a clear growth cone (Fig. 1B). In contrast, oculomotor leading processes are threadlike with no significant enlargement or structure at the tip (Fig. 1C). To investigate the molecular composition of the oculomotor leading processes, we performed immunocytochemistry for various cytoskeletal proteins associated with either tangential migration, e.g. TAG-1 (Yee et al., 1999) or process formation, e.g. drebrin (Mizui et al., 2005). This was performed at HH stage 28, which is when the first ventromedial cells separate from the nucleus (Chilton and Guthrie, 2004). In direct contrast to PCN leading processes (Yee et al., 1999), those of oculomotor neurons do not express TAG-1 but do express GAP-43 along their length and neurofilament heavy chains at the base; drebrin was the only protein with a significant distal localisation suggesting that its propensity to induce cell protrusions could be utilised for the generation of leading processes (Fig. 1D–F).

### Loss of drebrin blocks leading process formation and neuronal migration

**Supplementary Fig. S1:**
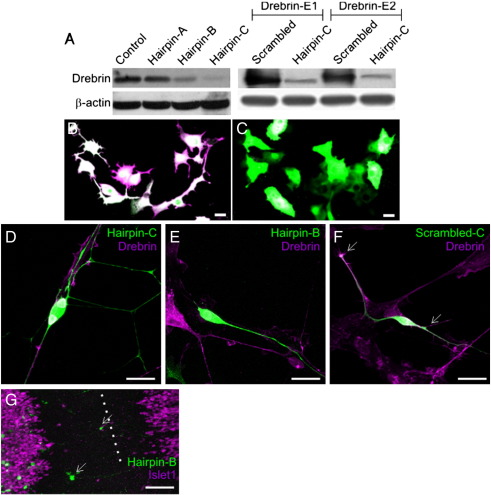
Validation of drebrin shRNA.

**Fig. 2:**
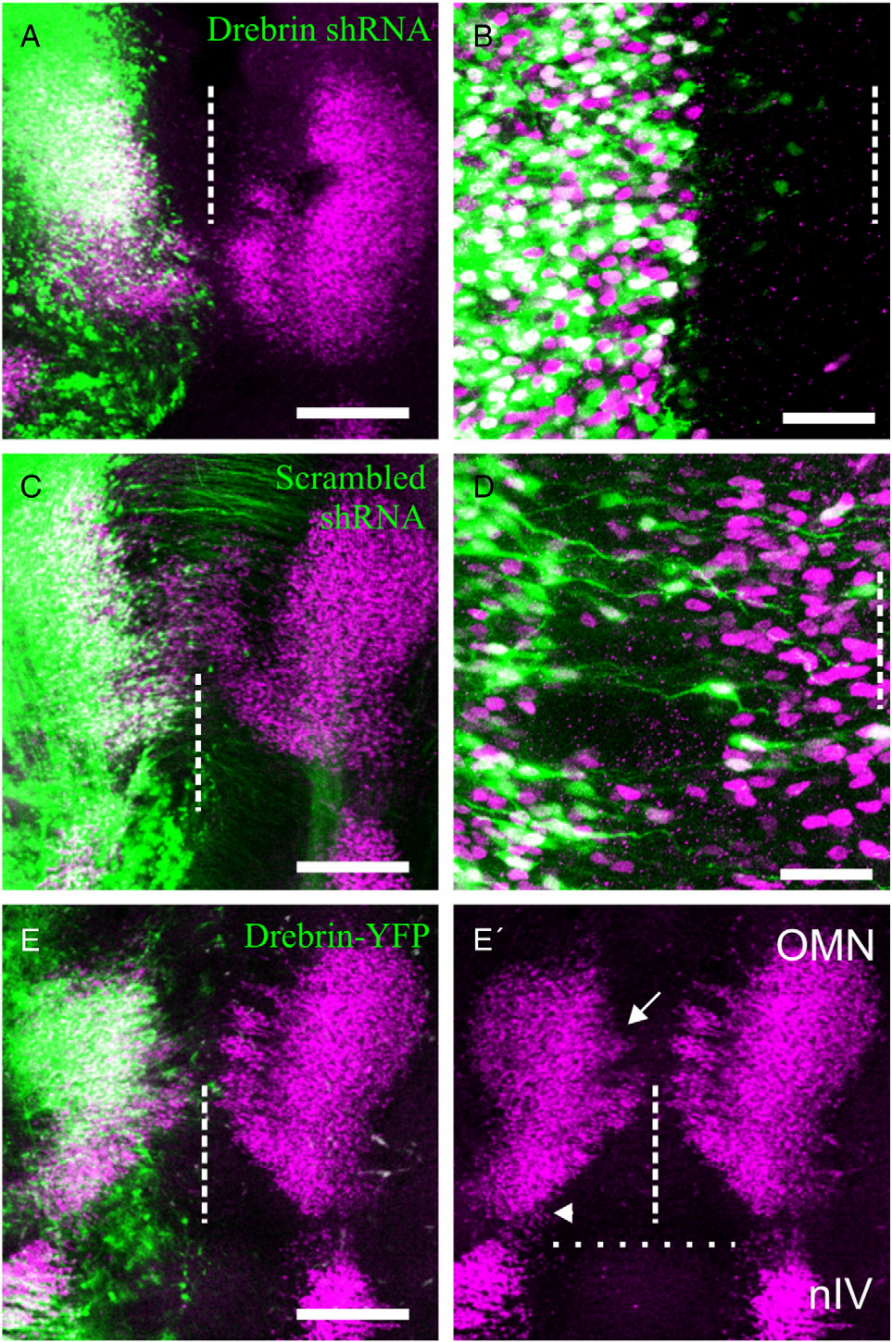
Drebrin is required for oculomotor neuron migration. (A) Electroporation of drebrin shRNA (green) blocks oculomotor migration and (B) leading process formation. (C,D) Electroporation of the scrambled sequence (green) had no effect. (E) Misexpression of drebrinE2-YFP (green) causes a loss in oculomotor neurons migrating to the midline (arrow) and a caudal expansion of the nucleus (arrowhead), reproduced in E′ with green channel omitted for clarity. Longitudinal view of whole mount preparations, motor neurons labelled with Islet-1 (purple), dashed line indicates midline, dotted line indicates midbrain-hindbrain boundary. OMN = oculomotor nucleus, nIV = trochlear nucleus. Scale bar = 200 μm (A,C and E), 50 μm (B and D).

In order to test the requirement for drebrin in the production of oculomotor leading processes, we generated short hairpin RNA (shRNA) within a plasmid vector to block its expression using *in ovo* electroporation (Bron et al., 2004). The efficiency of knockdown was confirmed by Western blotting of 3 T3 fibroblast cells – which do not express endogenous drebrin – co-transfected with the drebrin shRNA and chick drebrin cDNA, using a well-characterised antibody raised against chick drebrin (Shirao and Obata, 1986). Transfection with chick drebrin produced a single band which disappeared when drebrin shRNA was co-transfected (Supplementary Fig. S1). Furthermore, cells expressing the drebrin shRNA did not display the large filopodial protrusions normally induced by transfection with drebrin (Shirao et al., 1994). Finally, expression of the hairpins in chick embryonic dorsal root ganglion neurons knocked down endogenous drebrin as assessed by fluorescent immunocytochemistry (Supplementary Fig. S1). The sequence of the most effective hairpin was scrambled and used as a control (Supplementary Fig. S1). Drebrin shRNA was electroporated into the midbrain at HH stage 10–12 to target the oculomotor nucleus and the embryos collected at HH stage 29 when these neurons are normally reaching the midline (Chilton and Guthrie, 2004). In all embryos examined, the absence of drebrin blocked the formation of leading processes and migrating cells were not observed (n = 15; Fig. 2A and B), supporting our hypothesis that drebrin is required for this to occur. In contrast, oculomotor neurons electroporated with scrambled shRNA (n = 6; Fig. 2C, D) or GFP (n = 6) control vectors displayed normal leading processes and migratory behaviour. These data demonstrate that drebrin is required for the formation of the leading process in these neurons and that in its absence they fail to migrate.

### Overexpression of drebrin isoforms induces aberrant migration

**Supplementary Fig. S2:**
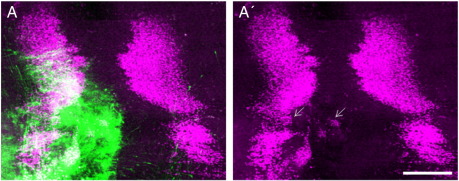
Overexpression of drebrinE1-YFP.

During early embryogenesis, two isoforms of chick drebrin, E1 and E2, are produced by alternative splicing of a 43 amino acid insert in E2 between residues 315 and 316 of E1. The expression of the E1 and E2 isoforms in the brain as a whole peak early and late respectively during embryogenesis prompting the suggestion that they are involved in distinct developmental processes (Shirao and Obata, 1986). We were unable to obtain shRNA that would selectively knockdown drebrin-E2 to test whether it alone is required for the generation of leading processes. We therefore asked what the relative effect of overexpressing drebrin isoforms would be upon migration. The two chick isoforms were cloned and fused to yellow fluorescent protein (YFP) for electroporation into the developing oculomotor nucleus. Overexpression of drebrin-E1 or E2 caused a reduction in the size of the column of migrating cells and a small but reproducible caudal elongation of the oculomotor nucleus (n = 18, E1; n = 12, E2; Fig. 2E, Suppl. Fig. S2). These results suggested that the levels of both isoforms have an influence upon migration but it was unclear whether they were specific to distinct aspects such as its initiation or direction.

**Fig. 3:**
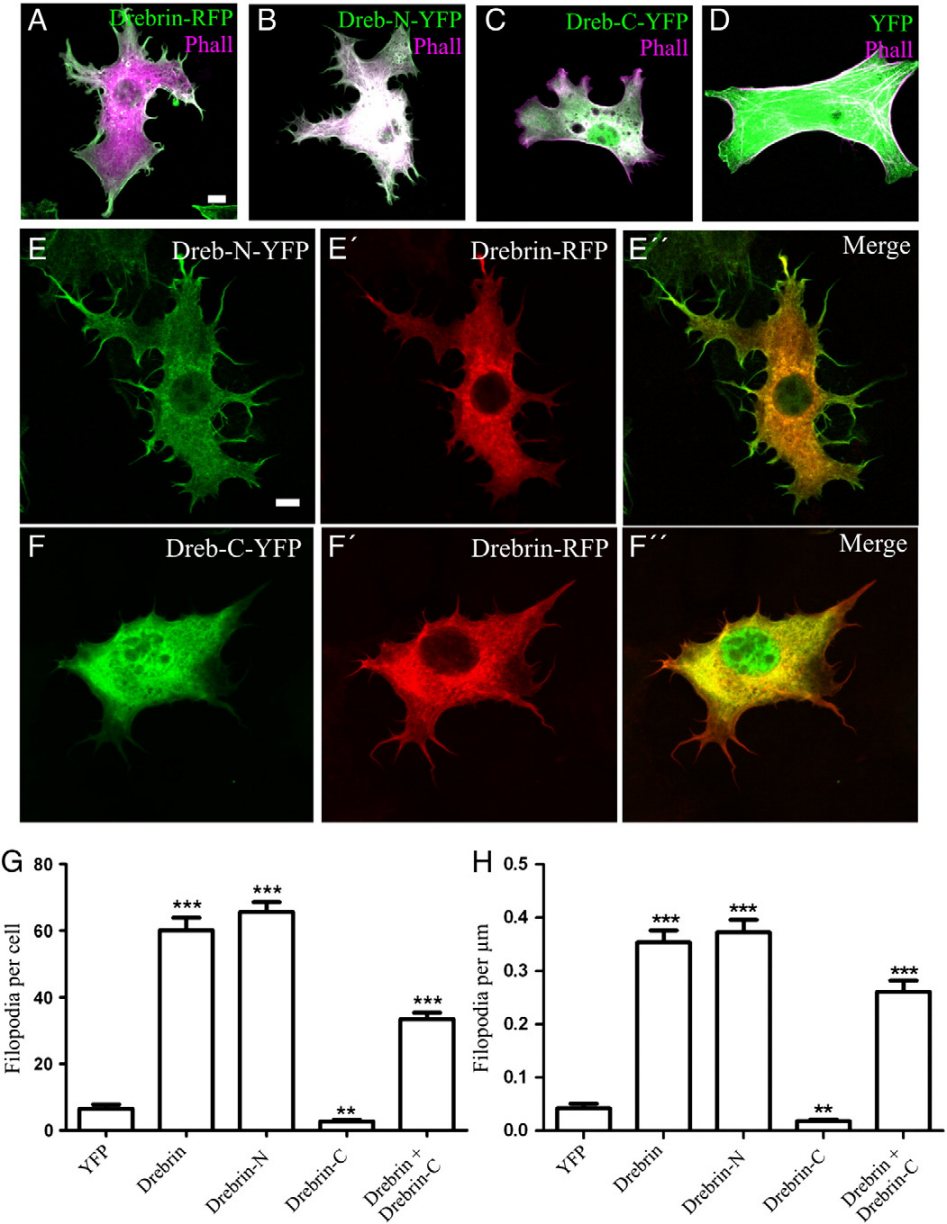
Analysis of drebrin domains required for the induction of spikes (A) Transfection of 3 T3 cells with chick drebrin-RFP induces filopodia as does drebrin-N-YFP (B) but not drebrin-C-YFP (C) compared to YFP alone (D). All constructs shown in green for consistency, cells counterstained with phalloidin (purple). (E) Drebrin-N-YFP displaces drebrin-RFP from filopodia when co-transfected. (F) Drebrin-C-YFP abrogates filopodial induction when co-transfected with drebrin-RFP. (G and H) Quantification of mean total number of filopodia per cell (G) and mean number of filopodia per micron of cell perimeter (H), 30 cells counted for each condition. Error bars indicate s.e.m. ** = p < 0.001, *** = p < 0.0001 compared to YFP (or to drebrin alone for drebrin + drebrin-C), unpaired *t*-test. Scale bar = 10 μm (A–F).

Leading process formation and cellular translocation are likely to require different modifications of the actin network and previous work has shown that domains within drebrin have differing affinities for actin-binding as opposed to actin remodelling (Hayashi et al., 1999). We therefore tested whether drebrin domains would have specific effects on migration. The N-terminal half of drebrin is highly conserved across vertebrate species and contains an ADF-cofilin homology domain followed by putative coiled-coil and helical regions within which the actin-binding domain is believed to lie (Grintsevich et al., 2010). The C-terminal half shows greater divergence and bears no homology to known protein domains apart from a Homer-binding motif (Dun and Chilton, 2010). We constructed chick drebrin truncations tagged with fluorescent proteins and quantified their ability to induce protrusions in transfected 3 T3 fibroblast cells. Fixed cells were also labelled with fluorescently-conjugated phalloidin to enable the quantification of the number and density of spikes. As expected, full-length chick drebrin-mCherry induced a greater number of spikes both in terms of total spikes per cell and spikes per unit length of cell perimeter (Fig. 3A, G and H). A truncated form, containing the ADF-cofilin homology domain and the coiled-coil and helical regions, terminating at amino acid 315 that we termed drebrin-N, did likewise (Fig. 3B, G and H). Drebrin-N did not induce more numerous spikes than full-length drebrin, however it was able to displace it from protrusions when co-transfected (Fig. 3E). The C-terminal half, comprising the rest of the protein and named drebrin-C, suppressed spike formation (Fig. 3C) compared to YFP (Fig. 3D, G and H) but when co-transfected with full-length drebrin or drebrin-N it abrogated spike formation (Fig. 3 F, G and H).

**Fig. 4:**
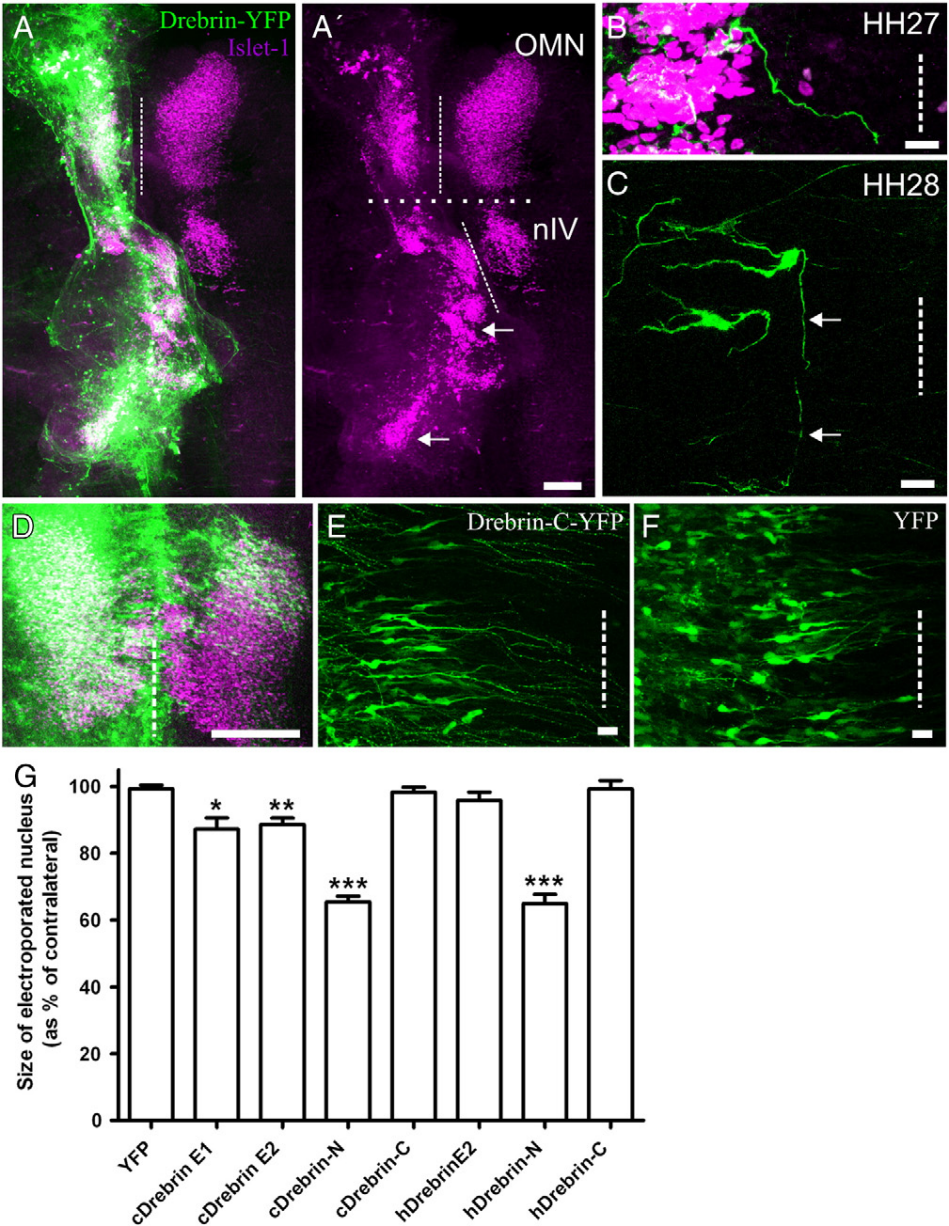
Changes in drebrin expression levels and activity lead to aberrant neuronal migration. (A) Misexpression of drebrin-N-YFP (green) causes aberrant migration of motor neurons into the hindbrain (arrows), reproduced in A′ with green channel omitted for clarity. (B) The leading process of oculomotor neurons misexpressing drebrin-N-YFP (green) meander and begin to turn from the midline at HH stage 27. (C) At HH stage 28, they have turned and grown caudally and away from the midline (arrows). (D) Misexpression of drebrin-C-YFP (green) has no effect on oculomotor neuron migration although the leading processes have a beaded appearance (E) compared to YFP alone (F). Longitudinal view of whole mount preparations, motor neurons labelled with Islet-1 (purple), dashed line indicates midline, dotted line in A′ indicates midbrain-hindbrain boundary. OMN = oculomotor nucleus, nIV = trochlear nucleus. (G) Quantification of reduction in oculomotor nucleus size as a result of aberrant migration following misexpression of chick (cDrebrin) and human (hDrebrin) constructs. Error bars indicate s.e.m. * = p < 0.05, ** = p < 0.001, *** = p < 0.0001 compared to YFP, unpaired *t*-test. Scale bar = 200 μm (A,D), 20 μm (B,C,E and F).

We next electroporated drebrin-N-YFP and drebrin-C-YFP into the oculomotor nucleus to examine whether the ability to induce or suppress spike formation could be related to the generation of leading processes. Overexpression of drebrin-N had a dramatic effect on oculomotor development; in none of the HH stage 29 embryos analysed (n = 8) were any Islet1/2-positive cells from the electroporated side observed across the midline. Instead, clusters of them migrated caudally into the hindbrain, towards the ipsilateral trigeminal nucleus (Fig. 4A). Despite crossing the midbrain–hindbrain and rhombomere boundaries, none of the ectopic neurons were observed to cross the midline. In order to examine the development of this phenotype from the onset of migration, we collected electroporated embryos from HH stages 27 and 28. At stage 27, electroporated cells had already extended processes but these took a meandering route towards the midline (Fig. 4B). Twelve hours later, at stage 28, the leading process had reached the midline and made a sharp caudal turn, pre-empting the trajectory of the aberrantly migrating cells (Fig. 4C). This result shows that, in addition to being required for the formation of oculomotor leading processes, drebrin plays a role in their ability to respond to environmental cues and navigate accurately. No defects were seen in embryos electroporated with drebrin-C (n = 7); electroporated neurons crossed the midline and remained within the midbrain (Figs. 4D and E). The leading processes grew as parallel fibres directly towards the midline although they did appear to contain varicosities when compared to YFP controls (n = 5; Fig. 4F). The ectopic Islet1/2 + cells produced by drebrin-N are almost certainly of oculomotor origin, with the possibility of a minor contribution from the trochlear nucleus, there are normally no other Islet-positive cells within the electroporated area. To provide further evidence that the ectopic cells would normally have constituted the OMN, we quantified the effect on it by expressing the size of the electroporated nucleus within the midbrain (identified visually by the morphological landmarks of the midbrain-hindbrain border and the midline) as a fraction of the unelectroporated one for each construct (Fig. 4G). This showed that drebrin-N significantly reduced the size of the nucleus by a mean of 35%; drebrin-E1 and E2 also caused significant but more modest reductions of 13% and 11% respectively whilst neither drebrin-C nor YFP had any effect. Therefore, based on the reduction in OMN size and the way in which the leading process reorientation pre-empts the caudal migration, we believe the evidence is strongly in favour of OMN cells that over-express drebrin leaving the midbrain.

In humans, mice and rats only one embryonic drebrin isoform has been isolated, drebrin-E, which is orthologous to chick drebrin-E2. However, given the high amino acid sequence identity of the region corresponding to drebrin-N (83% chick:human), they would be predicted to have the same effect on migration. Indeed, electroporation of human drebrin-N-YFP (amino acids 1-315) produced a comparable reduction in oculomotor nucleus size (n = 15; Fig. 4G) and the same migration defects. Chick and human drebrin-C show much less sequence identity (42%, the majority of which occurs in the 60 terminal residues) and the latter had no effect on migration (n = 13) but interestingly, neither did the full-length human drebrin-E2 (n = 11; Fig. 4G) suggesting that the exact sequence of the C-terminus is important for specifically regulating the overall activity of drebrin. Overall, the effects of misexpressing truncated forms of both chick and human drebrin show that the ability to induce leading processes lies within the drebrin-N region but that this is normally modulated by intra- or intermolecular interactions mediated by the C-terminus.

### Drebrin regulates axon growth cone morphology but is not absolutely required for axonogenesis or axon guidance

**Fig. 5:**
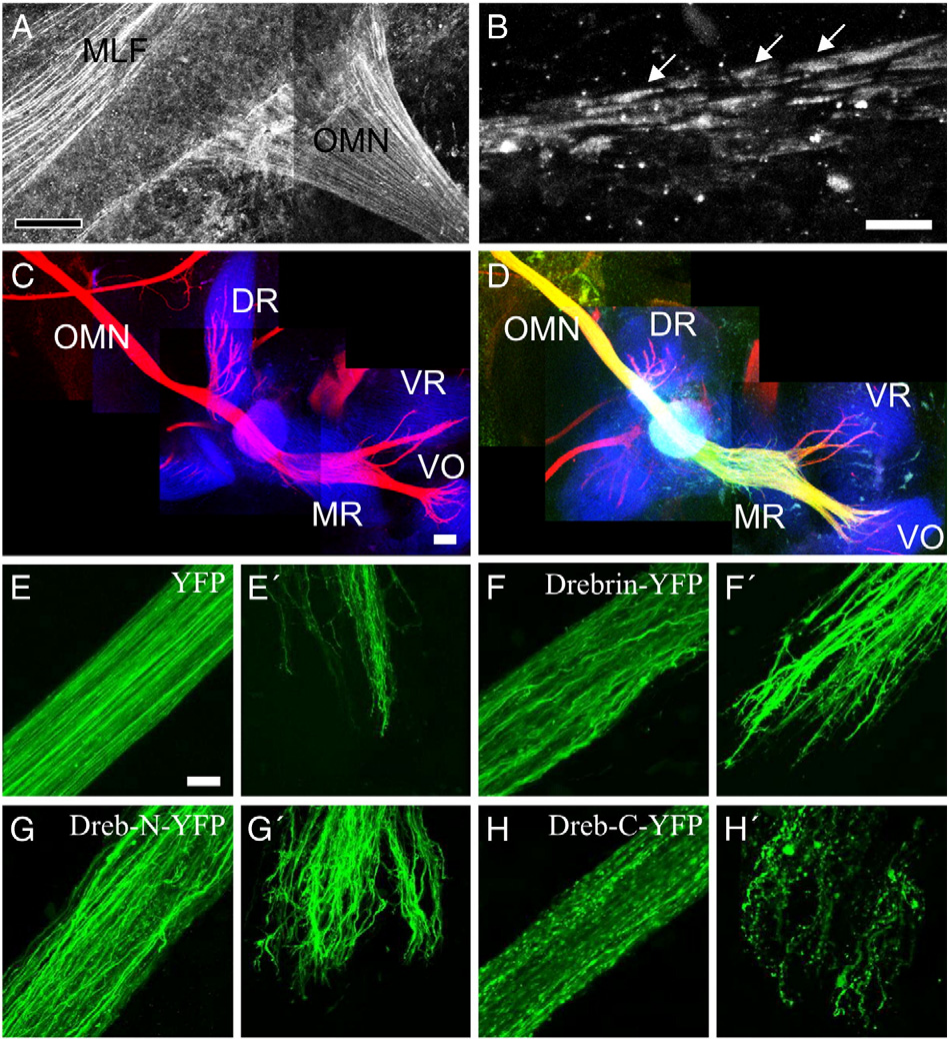
Drebrin regulates oculomotor axon morphology but does not affect pathfinding (A and B) Immunolabelling of endogenous drebrin in the proximal oculomotor nerve and axons of the medial longitudinal fasciculus (A) and in growth cones at the distal end of the oculomotor nerve (arrows, B). (C and D) Oculomotor neurons expressing drebrin shRNA (green) still form axons which reach their target muscles. Control, unelectroporated side (C), electroporated side (D). All nerves labelled with 3A10 antibody (red), muscles labelled with Alexa647-conjugated alpha-bungarotoxin (blue). OMN = oculomotor nerve, MLF = medial longitudinal fasciculus, DR = dorsal rectus, MR = medial rectus, VR = ventral rectus, VO = ventral oblique muscle. (E–H) Oculomotor axons and growth cones misexpressing YFP (E and E′ respectively), drebrin-YFP (F and F′), drebrin-N-YFP (G and G′) and drebrin-C-YFP (H and H′). Whole mount preparations, scale bar = 100 μm (A), 5 μm (B), 200 μm (C and D), 50 μm (E–H).

The marked effect of drebrin on leading processes, in conjunction with its known involvement in neuritogenesis (Geraldo et al., 2008) suggested that axonogenesis would be similarly perturbed so we analysed the peripheral projection pattern of the oculomotor nerve which is highly stereotyped (Chilton and Guthrie, 2004). We used whole mount immunohistochemistry to confirm that drebrin is found endogenously within oculomotor axons. Clear staining was visible at HH stage 27 in the proximal end of the oculomotor nerve where it exits the midbrain (Fig. 5A). Due to the depth of tissue, it was harder to examine the distal end, nevertheless at high magnification, endogenous drebrin was observed at axon tips in structures resembling growth cones (Fig. 5B). Drebrin shRNA completely blocked leading process formation yet in these same neurons, oculomotor axons were still produced and followed their normal route to the extraocular muscles (Fig. 5C and D). Whilst we cannot be certain that correct topographic mapping still occurs, the gross pattern of connectivity is indistinguishable from the wildtype. Therefore, the function of drebrin is compartmentalised within these neurons to perform a specific role in leading process initiation and orientation without significantly impinging on axonal extension or pathfinding.

**Fig. 6:**
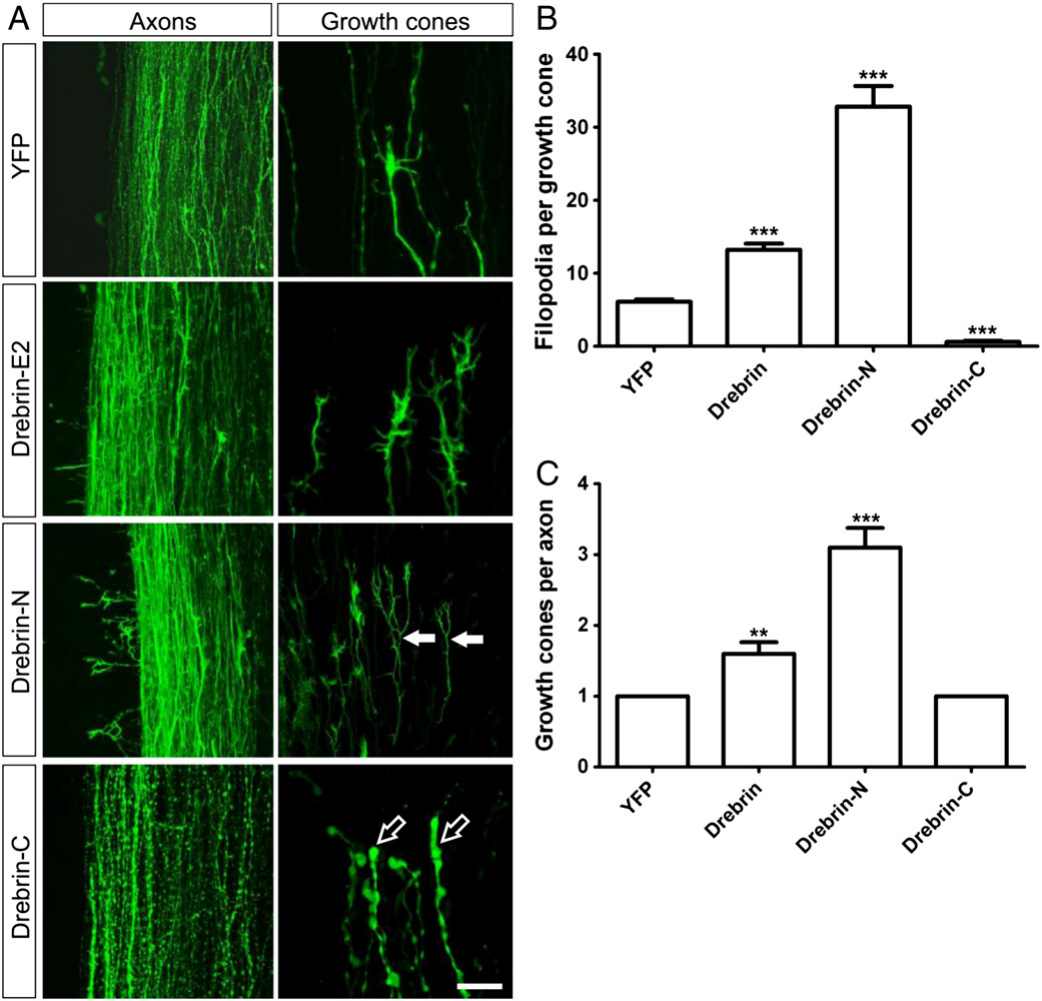
The actions of drebrin on axon morphology and pathfinding are also distinct within the medial longitudinal fasciculus (A) Representative images of the effect of electroporation of YFP-tagged drebrin constructs (labelled rows) into the medial longitudinal fasciculus (MLF) which leads to changes in axon (left hand column) and growth cone (right hand column) morphology, compared to YFP alone. Most notably, drebrin-N induces axon collaterals and axonal branching (filled arrows) whilst drebrin-C produces clubbed growth cones (outline arrows). Scale bar = 25 μm. (B and C) Quantification of changes in number of filopodia per growth cone (B) and number of growth cones per axon (C) following misexpression of drebrin-YFP constructs in the MLF. Error bars indicate s.e.m., 10 axons/growth cones counted per construct. ** = p < 0.01, *** = p < 0.0001 compared to YFP; unpaired *t*-test (B), Mann–Whitney test (C).

Overexpression of drebrin did affect axon and growth cone morphology but the overall trajectory of growth appeared grossly unaffected. After electroporation with YFP, the oculomotor nerve grows as a thick fascicle of straight axons with a frayed tip as individual growth cones diverge (Fig. 5E). Full-length drebrin produced undulations along the axons and the growth cones appeared thicker and enlarged (Fig. 5F), this was even more apparent following electroporation with drebrin-N (Fig. 5G). Drebrin-C did not cause axons to appear sinusoidal; they grew straight but with varicosities along their length (Fig. 5H), similar to its effect in leading processes; this was particularly apparent at the distal end. Clear visualisation of oculomotor growth cones is difficult due to the depth of tissue so we examined a nearby tract, the medial longitudinal fasciculus (MLF) which expresses drebrin (Fig. 5A) and in which the appearance of growth cones can be better discerned. The effect of drebrin on axon morphology was the same as observed in the oculomotor nerve (Fig. 6A). MLF growth cones produced an increased number of filopodia and the axons split into multiple growth cones when overexpressing full-length drebrin (13.2 ± 0.9 filopodia per growth cone; 1.6 ± 0.2 growth cones per axon; n = 10) or drebrin-N (32.8 ± 2.8 filopodia per growth cone; 3.1 ± 0.7 growth cones per axon; n = 10) compared to YFP (6.1 ± 0.3 filopodia per growth cone; 1.0 ± 0.0 growth cones per axon; n = 10) in which each axon had one growth cone (Fig. 6B and C). Conversely, drebrin-C (0.6 ± 0.2 filopodia per growth cone; 1.0 ± 0.0 growth cones per axon; n = 10) made growth cones become small and club-shaped with varicosities along the axon shaft (Fig. 6). These data provide a strong parallel between the induction of spikes *in vitro* and the generation of axon morphology *in vivo*.

## Discussion

The mechanics underlying neuronal migration are poorly understood and this study will help to advance their elucidation and potentially the aetiology of a number of human neurological disorders characterised by migration defects (Valiente and Marin, 2010). We have demonstrated a requirement for the actin-binding protein drebrin for the correct migration of oculomotor neurons. Drebrin is necessary at two stages of migration: firstly for the formation of the leading process and the initiation of migration; secondly for the correct orientation and navigation of the process and thus also the trajectory of the cell body. The ability of neurons to maintain functional divisions across the cell are a prerequisite for generating and maintaining correct connections (Allen and Chilton, 2009). Our data show that the actions of drebrin can be compartmentalised within individual neurons to perform a spatially localised role and regulate leading process guidance without impinging upon axon guidance. Neuronal leading processes and axonal growth cones execute similar navigational programmes during development and information obtained from studying the latter has often been extrapolated to the former. The degree to which this is relevant in terms of common molecular mechanisms is unknown; recent data are beginning to demonstrate that despite their similarities, axon guidance and cell migration may have fundamental differences (Marin et al., 2010; Martini et al., 2009). Studying the unique behaviour of this population of oculomotor neurons provides a means to address this question.

The relative roles of the two embryonic drebrin isoforms remain to be established, although there is a long-standing hypothesis that the control of their splicing during development reflects involvement in distinct stages of brain maturation (Shirao and Obata, 1986). Our data did not distinguish between the two isoforms in terms of their effect on migration when misexpressed *in ovo*. The carboxy terminus of drebrin seems to dampen its activity: our co-transfection experiments showed that it could abrogate the spine-inducing activity of the full-length protein. Furthermore, drebrin-N displaces full-length drebrin from filopodial protrusions which supports the idea that the presence of the carboxy terminus modulates the ability to bind and remodel actin filaments. This is in agreement with other work which has shown that binding of drebrin to the chemokine receptor CXCR4 is mediated by the N-terminus of drebrin and happens with greater affinity in the absence of the C-terminus (Perez-Martinez et al., 2010). The C-terminus of drebrin may act in a general auto-inhibitory manner or mediate the recruitment of secondary factors. We favour the second hypothesis because if it was a direct inhibition then overexpression of drebrin-C would be expected to produce a phenotype closer to the effect of drebrin shRNA by blocking the function of endogenous drebrin. However, drebrin-C did not have an inhibitory effect on migration *in ovo* only on morphology, implying that its ability to suppress spike formation in fibroblasts more closely models the effect of drebrin on neuronal shape rather than on navigation. Furthermore, full length human drebrin (unlike human drebrin-N) had no effect on migration in the chick, supporting the notion that specific binding to accessory proteins is necessary.

Tangential neuronal migration is generally distinguished from radial migration by the absence of an underlying scaffold and a trajectory parallel to the ventricular surface (Lambert de and Goffinet, 2001). However, within those populations of neurons that migrate tangentially a number of distinct features are becoming apparent (Marin et al., 2010). Foremost among these is the presence of branched leading processes, exhibited by cortical neurons among others. The generation of new branches in response to extracellular cues allows rapid changes in direction without the need to re-orient the existing process (Martini et al., 2009). Oculomotor leading processes do not produce any branches, possibly these are not required for their relatively short journey. However, after drebrin overexpression, we observed reorientation of the entire leading process rather than increased branching. Interestingly, the opposite occurred in the axons, these produced additional branches but the direction of growth was not altered. This suggests the existence of two separate growth mechanisms that can be selectively employed either by neuronal populations or within one cell. The co-ordination of axonogenesis with neuronal migration and wider programs of differentiation is a problem faced by all developing neurons but whether these aspects can run in parallel or require sequential regulation is unclear (Marin et al., 2010). The functional interdependence of these two processes is demonstrated by our results: Although the axonal projections appear grossly normal, either the ectopic neurons may not survive the pruning that accompanies neuromuscular maturation or the cell bodies may not receive appropriate inputs from upper motor neurons. In either case, the resultant oculomotor system would not function correctly. A salient feature of oculomotor neurons is that the leading process and axon are physically distinct protrusions (Chilton and Guthrie, 2004; Puelles-Lobez et al., 1975), unlike other tangentially migrating neurons, such as the PCN in which the leading process becomes the axon (Bloch-Gallego et al., 2005). Thus in oculomotor neurons it would be more straightforward to separate the two mechanisms, for instance by regulating the passage of necessary signalling components into the axon (Allen and Chilton, 2009). Different repertoires of cytoskeletal proteins may be another distinction between classes of migrating neurons, for example, we show here that oculomotor leading processes contain GAP-43 but not TAG-1, the opposite to the PCN (Yee et al., 1999).

The cytoskeleton is a fundamental factor in neuronal migration, it requires an elaborate interplay between the actin and microtubule networks and the molecules that regulate them (Kawauchi and Hoshino, 2008). Drebrin is primarily an actin-binding protein but also binds to EB3 on microtubule tips (Geraldo et al., 2008) and is therefore well-placed to co-ordinate actin-microtubule interactions. The balance between the two may underlie the different modes of process extension, for example regulation of microtubule turnover by LIS1 is linked to the generation of branched leading processes (Gopal et al., 2010). Alternatively, actin and microtubules may predominate during different phases of migration. F-actin is required within the leading process for somal translocation to occur, in conjunction with myosin II activity and microtubule stabilisation (Asada and Sanada, 2010; He et al., 2010). In the absence of drebrin, leading processes might fail to form due to insufficient formation of F-actin bundles within them or inadequate cross-linking to microtubules; blocking microtubule dynamics is known to impair directional neuronal migration (Baudoin et al., 2008). Directional migration stems from varied morphological and motile changes that result from the cumulative effect of many parameters such as the size, shape, orientation and spread of protrusions. Each of these can be affected by specific enzymatic regulators of actin and microtubules (Sakakibara and Horwitz, 2006) and future work will need to determine which of these modulate drebrin or serve as its effector. For example, drawing a parallel with our observations of drebrin, appropriate levels of Rac activity are needed to drive the correct protrusive activity, suppress aberrant branching and maintain directional migration (Pankov et al., 2005; Sakakibara and Horwitz, 2006).

## Conclusions

Our data show that drebrin, a protein known to modulate turnover of the neuronal cytoskeleton, plays a crucial role in directed motility by enabling leading process formation and subsequently its ability to correctly interpret external signals. Developing neurons often have to execute parallel programs of axonogenesis and migration which can place conflicting navigational demands upon the cell. This may be overcome by intracellular restriction of the actions of proteins within relevant cellular compartments.

The following are the supplementary materials related to this article.

## Experimental methods

### DiI labelling and immunohistochemistry

Fertilised hens’ eggs (Gallus gallus) were obtained from a recognised national supplier (Henry Stewart & Co. Ltd., Lincs.) and incubated in a humidified, forced-draft incubator at 38 °C until the desired stage; staging was based on Hamburger and Hamilton (Hamburger and Hamilton, 1992). Embryos were removed from the egg, decapitated and the head further dissected in PBS. A 0.1% (w/v) solution of 1,1′-didodecyl-3,3,3′,3′-tetramethylindocarbocyanine perchlorate (DiI-C_12_) in ethanol was injected into the dorsal rectus muscle and the tissue fixed in 4% paraformaldehyde at 37 °C for one week to allow the DiI to diffuse to cell bodies and leading processes. The midbrain was dissected out and cleared in a glycerol/PBS series before mounting in 50% glycerol/PBS.

For immunolabelling of the oculomotor leading processes and cell bodies, the whole head was fixed overnight at 4 °C in 4% paraformaldehyde, washed in PBS, equilibrated in 20% sucrose and then rapidly frozen in OCT embedding medium for cryosectioning. Immunocytochemistry was performed as previously described (Chilton and Guthrie, 2004), antibodies used were: anti-drebrin monoclonal (M2F6, Cambridge Bioscience), anti-GAP43 rabbit polyclonal (Abcam), anti-TAG1, anti-neurofilament heavy chain (Calbiochem) and AlexaFluor conjugated secondary antibodies and phalloidin (Invitrogen). The Islet-1 antibody (39.4D5) developed by Jessell and Brenner-Morton was obtained from the Developmental Studies Hybridoma Bank developed under the auspices of the NICHD and maintained by The University of Iowa, Department of Biology, Iowa City, IA 52242.

### Cloning of chick drebrin vectors

Total RNA was extracted from HH stage 32 chick brains using TRI Reagent (Applied Biosystems) and cDNA was synthesised by reverse transcription (M-MLV Reverse Transcriptase, Promega) using oligo-dT (n = 15) primers. To clone drebrin-E1 and E2, primers were designed and synthesised with *BamHI* and *EcoRI* restriction sites at the 5′ and 3′ ends respectively and DNA was amplified by PCR using *Pfu* polymerase (Promega). Primer sequences used were: 5′-attaggatccatggctggcgtcggcttcgc-3′ and 5′-atatgaattccaggccgcccccgaagctctc-3′. To clone N-terminal and C-terminal truncated forms, two other primers were used: 5′-attaggatccatgagcacccaggtggcagagccggcagcgactgagca-3′, 5′-tgtcctatggctgcccggcgagatggcgtc-3′ with chicken Drebrin E1 as the template. All PCR products were subcloned into the Zero Blunt TOPO vector (Invitrogen) and verified by sequencing. Full length Drebrin E1, E2 and truncations were subsequently excised by *BamHI* and *EcoRI* digestion and inserted into the corresponding sites of pclink-YFP or pclink-RFP (Geraldo et al., 2008; Peris et al., 2006).

Short hairpin RNA (shRNA) against chick drebrin were designed and constructed as described previously (Bron et al., 2004). The target sequence was:

Forward primer: 5′-gccaagatcgcagagttcttacacgtcaagaactctgcgatcttggcctttttt
Reverse primer: 5′-aaaaaaggccaagatcgcagagttcttgacgtgtaagaactctgcgatcttggcggcc

The scrambled sequence was:

Forward primer: 5′-tcactgatggtcgctaagattcaagagatcttagcgaccatcagtgagctttttt
Reverse primer: 5′-aaaaaagctcactgatggtcgctaagatctcttgaatcttagcgaccatcagtgaggcc

### Cell culture and spike quantification

Circular coverslips (13 mm diameter) were placed in a 6-well plate into which NIH 3 T3 cells were seeded and cultured in DMEM supplemented with 10% fetal bovine serum, penicillin and streptomycin (Sigma) at 37 °C and 5% CO_2_. Cells were transfected using Lipofectamine LTX in accordance with the manufacturer's instructions (Invitrogen). Thirty hours after transfection, cells were fixed with a mixture of 4% paraformaldehyde and 0.2% glutaraldehyde for 15 min at 37 °C. Fixed cells were washed three times with phosphate-buffered saline (PBS) and mounted in FluorSave (Calbiochem) for image capture on a Zeiss LSM510 confocal microscope. Z-stacks were compressed using an extended projection view and spike parameters quantified using ImageJ software.

### In ovo electroporation and whole mount immunohistochemistry

*In ovo* electroporation was performed at HH stage 10–12 as previously described (Miyake et al., 2008). To target the oculomotor nucleus, DNA was injected into the mesencephalic vesicle. Once they had reached the desired stage, embryos were removed from the egg and examined under epifluorescence to select those that had been successfully electroporated. These were then processed for whole mount immunohistochemistry of the oculomotor nucleus using a standard protocol (Chilton and Guthrie, 2004). YFP fluorescence was amplified using a rabbit polyclonal antibody against GFP (Invitrogen), nerves were labelled with 3A10 monoclonal antibody (Developmental Studies Hybridoma Bank, University of Iowa), muscles and ciliary ganglia were labelled with Alexa647-conjugated alpha-bungarotoxin (Invitrogen). Images were obtained with a Zeiss LSM510 confocal microscope. Several Z-series were captured, covering the entire field of interest. The individual series were then flattened into a single image for each location and combined into one image in Adobe Photoshop (Adobe Systems). To calculate oculomotor nucleus size, 3-D reconstructions of Z-stacks within the anatomically defined area were measured using Volocity software, after subtraction of background by applying an intensity threshold (PerkinElmer).
